# Visualizing the spatiotemporal map of Rac activation in bovine aortic endothelial cells under laminar and disturbed flows

**DOI:** 10.1371/journal.pone.0189088

**Published:** 2017-11-30

**Authors:** Shuai Shao, Cheng Xiang, Kairong Qin, Aziz ur Rehman Aziz, Xiaoling Liao, Bo Liu

**Affiliations:** 1 Department of Biomedical Engineering, Faculty of Electronic Information and Electrical Engineering, Dalian University of Technology, Dalian, China; 2 Mathematical Information Technology, Faculty of Information Technology, Department of Math, University of Jyvaskyla. Jyvaskyla, Finland; 3 Department of Electrical and Computer Engineering, National University of Singapore, Singapore, Singapore; 4 Biomaterials and Live Cell Imaging Institute, Chongqing University of Science and Technology, Chongqing, China; University of California San Diego, UNITED STATES

## Abstract

Disturbed flow can eliminate the alignment of endothelial cells in the direction of laminar flow, and significantly impacts on atherosclerosis in collateral arteries near the bifurcation and high curvature regions. While shear stress induced Rac polarity has been shown to play crucial roles in cell polarity and migration, little is known about the spatiotemporal map of Rac under disturbed flow, and the mechanism of flow-induced cell polarity still needs to be elucidated. In this paper, disturbed flow or laminar flow with 15 dyn/cm^2^ of average shear stress was applied on bovine aortic endothelial cells (BAECs) for 30 minutes. A genetically-encoded PAK-PBD-GFP reporter was transfected into BAECs to visualize the real-time activation of Rac in living cell under fluorescence microscope. The imaging of the fluorescence intensity was analyzed by Matlab and the normalized data was converted into 3D spatiotemporal map. Then the changes of data upon chemical interference were fitted with logistic curve to explore the rule and mechanism of Rac polarity under laminar or disturbed flow. A polarized Rac activation was observed at the downstream edge along the laminar flow, which was enhanced by benzol alcohol-enhanced membrane fluidity but inhibited by nocodazole-disrupted microtubules or cholesterol-inhibited membrane fluidity, while no obvious polarized Rac activation could be found upon disturbed flow application. It is concluded that disturbed flow inhibits the flow-induced Rac polarized activation, which is related to the interaction of cell membrane and cytoskeleton, especially the microtubules.

## Introduction

Usually, atherosclerosis occurs at the branch points and curved regions of the arterial tree, where the blood flow remains unsteady and the shear stress shows marked spatial and temporal variations [1]. This should be due to the variations of flow patterns-induced functional differences of endothelial cells (ECs), such as migration and proliferation, in vascular system [2]. ECs orient prominently parallel to the direction of blood flow in the straight part of the arterial tree, while little orientation of ECs is found at the branch points and curved regions where flow patterns are more disturbed with no clear forward direction [3]. In vitro experiments have also proved the relationship between the flow patterns and ECs orientation. Laminar fluid shear would cause cell deformation along the flow direction [4], while Bovine aortic endothelial cells (BAECs) subjected to disturbed flow have a morphology and random orientations similar to those under static condition [5].

Rac is a group of plasma membrane bound proteins and plays important roles in controlling membrane ruffling and the formation of lamellipodia [6]. It is activated significantly in BAECs upon shear stress application, and then participates in the cell elongation and directionality of cell movement [7]. Other researchers have deeply explored that Rac was primarily activated at the leading edges of cells along the flow direction, or inhibited at the edges facing to the flow [8–10]. This polarity of Rac activation will promote the lamellipodium extension and the formation of new adhesions in migrating cells, and finally results in cell orientation [11]. Therefore, Rac should be one of the key signal proteins in the shear stress-mediated ECs orientation, and its local activation under different flow patterns may determine the direction of cell polarity. However, no experimental report could be found on the spatiotemporal model of Rac activation upon disturbed flow application, and the mechanism of Rac activation upon flow application also needed to be elucidated.

In this study, the spatiotemporal model of Rac was examined in BAECs under disturbed or laminar flows, analyzed the image with a developed and implemented software package in MATLAB (Mathworks; Natick, MA), and further explored the mechanism of shear stress-induced Rac local activation. It may present new molecular-level insights into the pathogenic mechanism of atherosclerosis.

## Methods

### Cell culture and transient transfection

Before transfection, the high glucose of Dulbecco's modified Eagle medium (DMEM, GIBCO, Invitrogen, USA) containing 10% fetal bovine serum, 2 mmol/l L-glutamine, 100 unit/ml penicillin and 100 mg/ml sodium pyruvate (GIBCO BRL) were used to culture BAECs isolated from bovine aorta(provided by local slaughterhouse) in a humidified CO_2_ incubator. According to the product specification, Lipofectamine 2000(Invitrogen) was used to transfect different DNA plasmids into cells[12]. Cells were starved in cell culture medium with 0.5% FBS for 36 hr for expressing various exogenous proteins. After that, cells were passed onto fibronectin-coated cover slips for 12 hr.

The fluorescent probe used in our experiment was a genetically-encoded PAK-PBD-GFP reporter containing a PAK domain to bind to active Rac and transfected into BAECs to obverse the real-time translocation of active Rac in living cell[13–15].

### Flow systems

**Fig 1:**
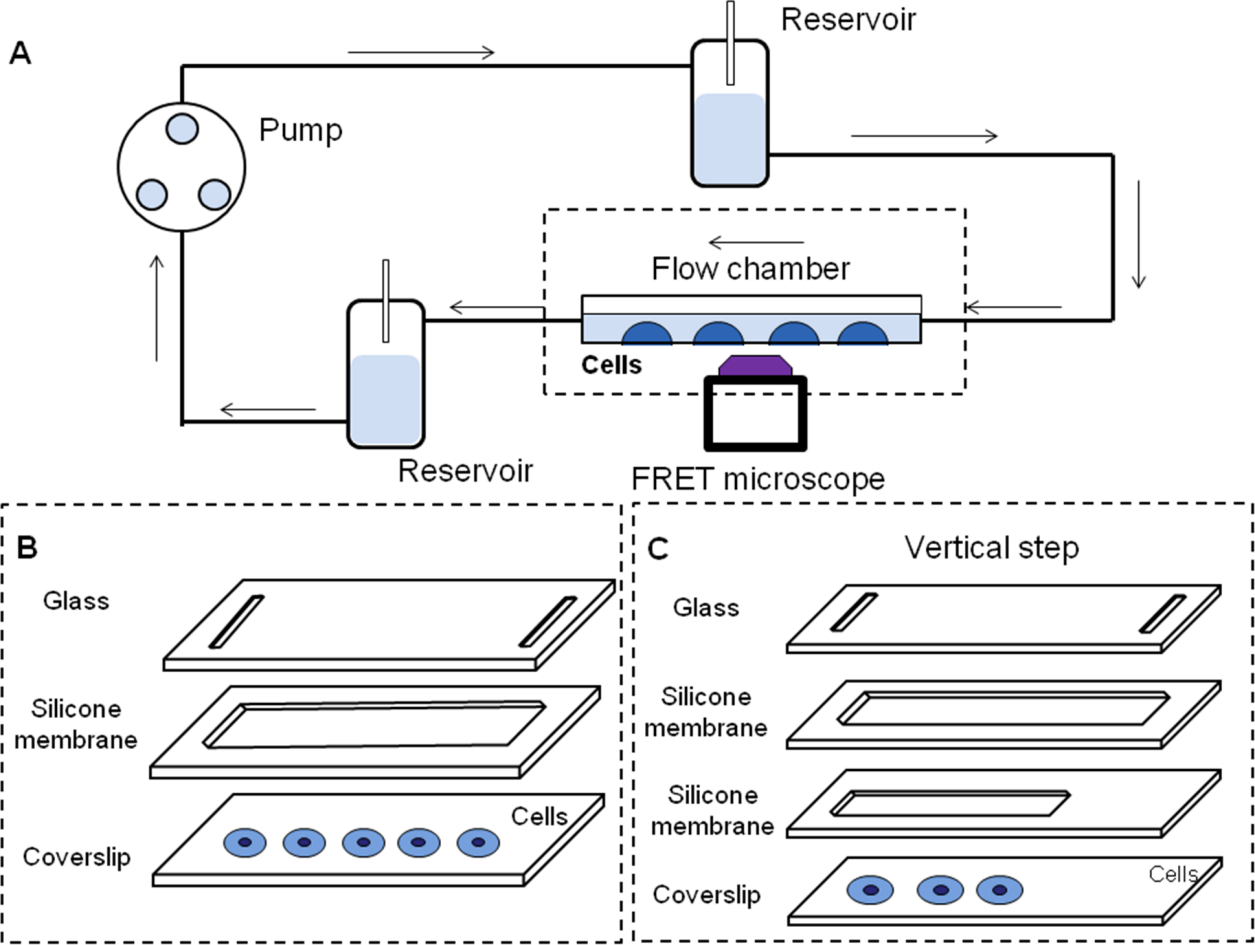
The system providing laminar flow or disturbed flow. (A) The whole system for providing different flows. (B) The structure in flow chamber to provide laminar flow contained a slip of glass, a silicone membrane with rectangular space and a coverslip. (C) The structure of vertical step to provide disturbed flow is consist of a slip of glass, two silicone membranes with different sizes of rectangular space and a coverslip.

As shown in Fig 1, a parallel-plate flow chamber was used to provide a laminar flow and a classic vertical-step, close to the entrance, was devised to impose the disturbed flow [16]. Silicone gasket with rectangular space was sandwiched between a glass slide, seeded with separated BAECs, and a cover glass. The laminar or disturbed flow in the flow channel was provided by one or two silicone gaskets respectively. The channel was 10 mm wide and the entrance was 0.25 mm high. The heights of the main channel for providing disturbed flow and the whole channel for laminar flow were both 0.5 mm. The total length of the flow chamber was 45 mm and the length of the entrance was 15 mm. In this flow system, flow patterns were confirmed by medium containing polystyrene particles which perfused the whole system and the region of disturbed flow in the chamber was marked out. The height difference between two reservoirs led to a hydrostatic pressure which induced the flow to which the cells were exposed. Laminar shear stress was set to 15 dyn/cm^2^ and the same mean flow was set in disturbed flow [17]. The conditions of the flow experiments were 37°C with 5% CO_2_ to maintain the pH at 7.4.

### Microscope image acquisition

All images were obtained only on isolated single cell under an inverted microscope (Nikon Eclipse Ti Se-ries, Ti-Fl Epi-fl/1) equipped with a cold CCD (Evolve512, Photometrics). Time lapse fluorescence images were acquired by MetaFluor 7.0 software (Universal Imaging) once after every 30 or 60 sec and arranged in chronological order beginning from 001.

### Image analysis

To analyze the fluorescence data rapidly, a software package in Matlab (Mathworks; Natick, MA) was developed. It contained two sections to allow the pre-treatment and fluorescence analysis respectively and allowed users to input the time of shear stress applied to cells. In both sections, the fluorescence intensity images were dyed by pseudo colors closer to red representing higher intensities and the pseudo colors closer to blue representing low intensities, respectively.

All the fluorescence images were regulated rightly according to the flow direction to ensure that the downstream region was to the left of the cell. Because the single cell was in migration upon laminar flow, the edge position needed to be identified in each image. The background was the average fluorescence intensity of the four corners of images and was subtracted before image quantification and analysis. The boundary, outlining the mask of the cell body, was enhanced by filtering speckle and close operation. The second module divided the cells both along the flow direction and the vertical direction into 50×50 parts averagely. The temporal changes of the fluorescence percentage in each part were calculated and saved as numbered mat file. The first image after shear stress application was named as I_1_, the second was I_2_ and the last one was I_30_. Subtraction was used to eliminate the fluorescent interference of the cellular nuclear or biosensor itself. The specific solution is as below:

$$I=(I_{2}{\mathrm{-I}}_{1})+(I_{3}{\mathrm{-I}}_{2})+\ldots \ldots +(I_{30}{\mathrm{-I}}_{29})$$ (1)

**Fig 2:**
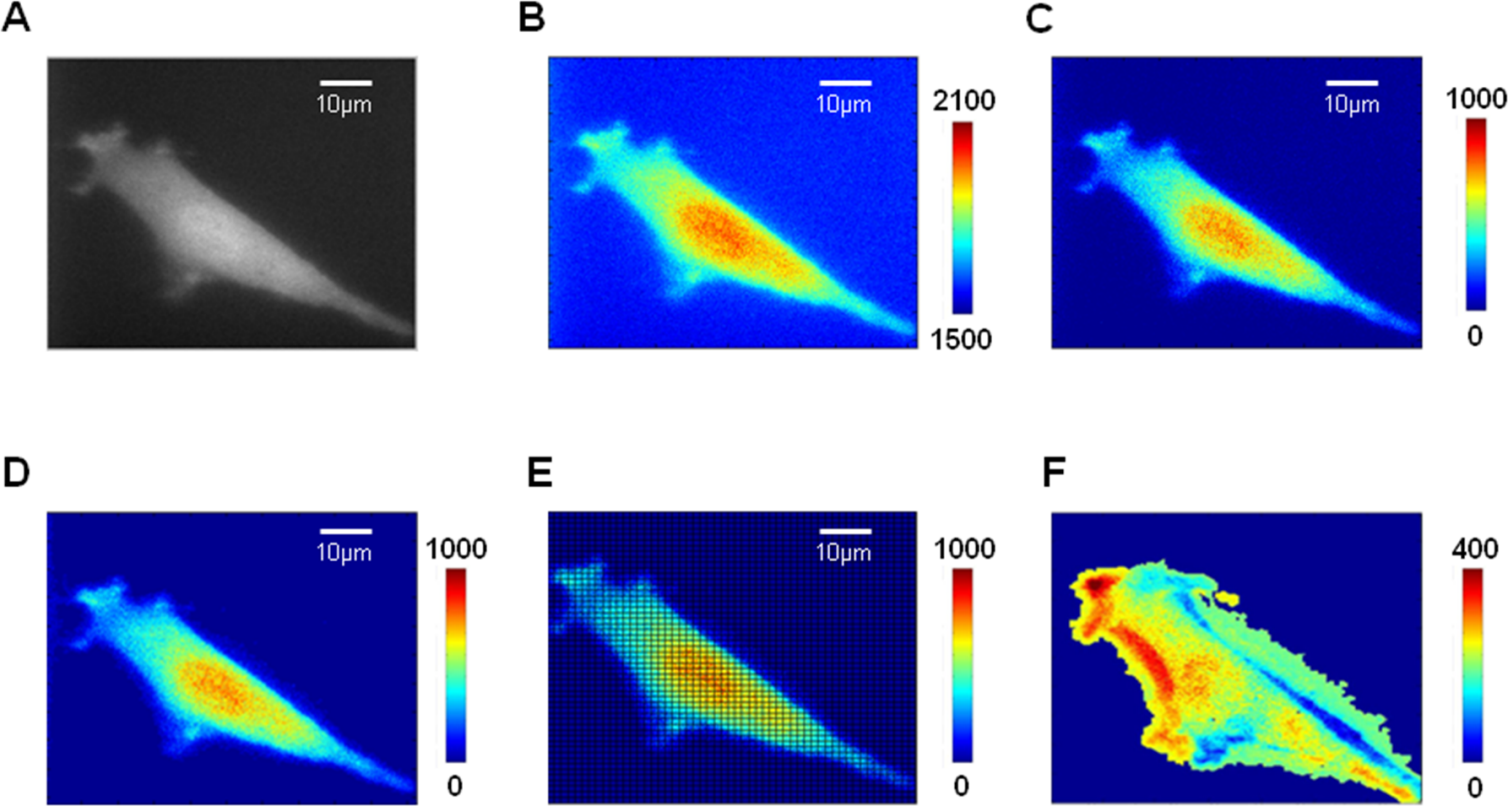
The diagrammatic sketch of image analysis. The original fluorescence image of BAECs (A) were added Pseudo color (B), then subtracted the background(C) and enhanced the edge of cell (D). After that, the image was divided into 50×50 parts (E) to calculate and eliminate the interference of nucleus (F).

Moreover, fluorescence matrix data of one sample at a certain time was compressed into a line along the direction of flow through projection. The specific method was calculating the percentage of fluorescence intensity in each column (columns are arranged along the direction of flow) from the whole cell. Percentages were arranged in chronological sequence to describe the spatiotemporal map of Rac activation by matrix. All fluorescence percentage data were normalized to a same basal level to avoid individual differences. Average matrix of samples in a same group were calculated and drawn into three-dimensional images by the Matlab function of ‘imagesc’. The fifty parts of the cell were numbered from 1 to 50 corresponding to the last part of the downstream and the first part of upstream, respectively. The diagrammatic sketch of image analysis is shown in Fig 2.

### Mathematical model analysis

All the fluorescence percentage data was normalized by their basal levels before stimulation in the same cell. In order to describe the level of the polarity more precisely, the normalized fluorescence percentage along the flow was fitted with logistic curve as follows,

$$y=\frac{A_{1}-A_{2}}{1+e^{(x-X_{0})/D}}+A_{2}$$ (2)

where x is a spatial variable indexed by the serial number of each part divided along the flow direction, y is the fitting value of the normalized actual fluorescence percentage, and A_1_, A_2_, X_0_ and D denote the maximum, the minimum, the inflection point and the parameter related to the slope, respectively. Among all the logistic parameters obtained by curve fitting, the absolute value of difference between A_1_ and A_2_, |A_1_-A_2_|, was related to the intensity of polarity. The slope of each point was calculated from the first derivative of the logistic curve. The point X' could be considered as the position where Rac polarity appears when the slope of X’ or (X’+1) was very close to zero (<0.005) but (X’-1) was not. The slope of the inflection point X_0_ was related to the parameter D as shown in Eq (3).

$$y^{\prime }\left(X_{0}\right)=\frac{A_{2}-A_{1}}{4D}$$ (3)

Statistical analysis for fitting parameters was performed by using a Student’s T-test function in the Excel software (Microsoft) to evaluate the statistical difference between groups. A significant difference was determined by the p-value (< 0.05).

## Results

### Disturbed flow inhibits the polarity of Rac

**Fig 3:**
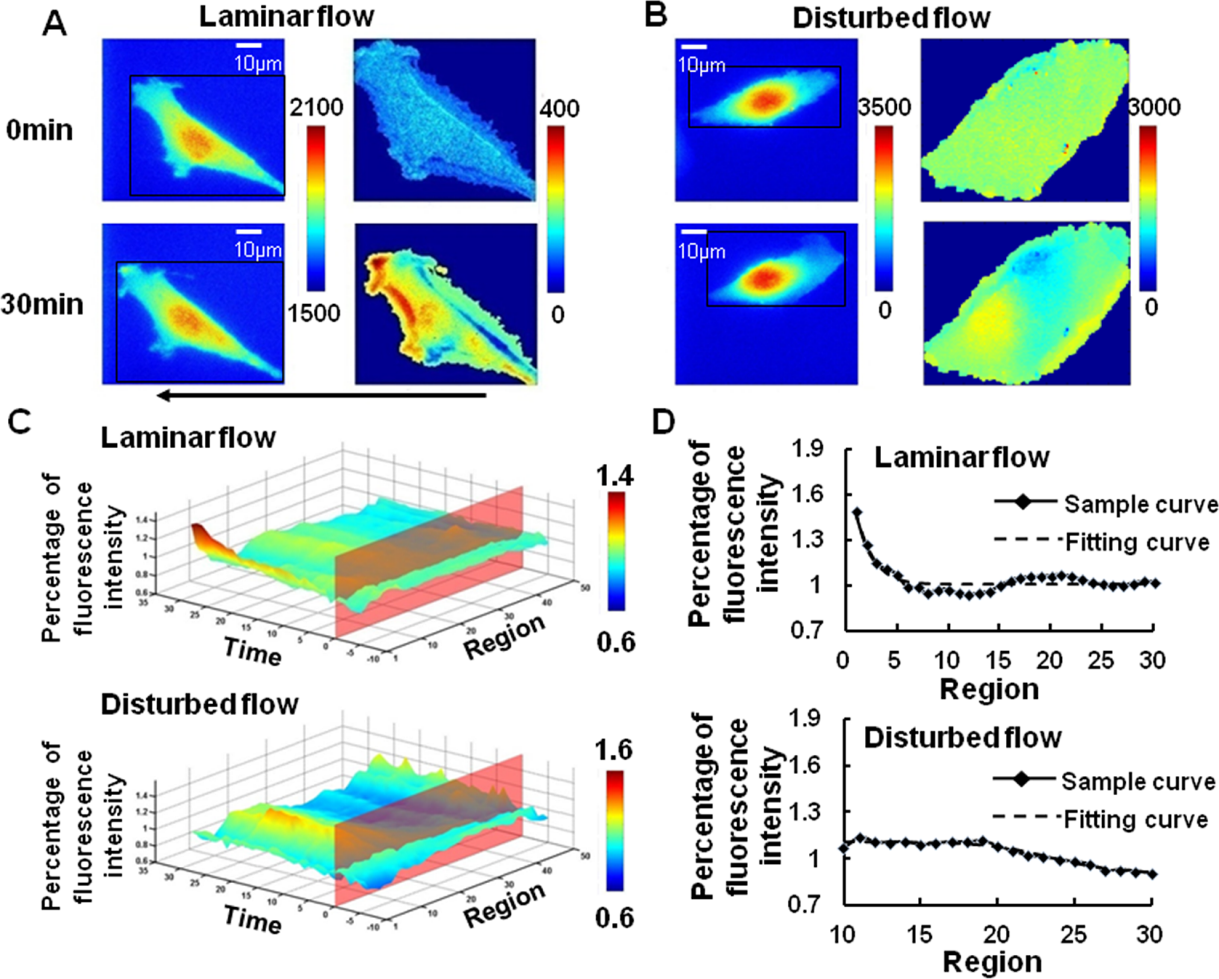
Polarized Rac activation in BAECs was caused by laminar flow but not disturbed flow. The image of BAECs transfected with PAK-PBD-GFP biosensor to represent the Rac activation (Left panel) upon laminar flow (A, n = 12) or disturbed flow (B, n = 5) application were dealt by our imaging analysis software (Right panel). The 3D spatiotemporal map (C) of averaged single cell images showed an obvious Rac activation at the downstream of BAECs from laminar flow group (Above), but not disturbed flow group (Below). After that, the data of the thirtieth minute from laminar flow (Above) and disturbed flow (Below) were fitted by logistic curve (D). The arrow indicated the main flow direction.

The PAK-PBD-GFP translocation at the downstream edge of the cell was observed gradually upon 15 dyn/cm^2^ of laminar flow application for 30 min. However, after 30 min of disturbed flow stimulation, with the same average shear stress 15 dyn/cm^2^, the fluorescence was found distributed uniformly in the whole cell (Fig 3A and 3B). After further quantification by our software, as shown in Fig 3C and 3D, no obvious fluorescence accumulation could be found at downstream edge of the spatiotemporal map, suggesting that there was not much activated Rac at downstream part.

Through fitting the data with logistic curve, the resulting parameters were applied in T-test to measure the differences between the two groups (Table 1). The result of T-test showed that X’, which was related to the starting position of Rac polarity, was much different from control. In addition, the range of the logistic curve, |A_1_-A_2_|, which described the intensity of the polarity, decreased. Together with the reduced slope, the two parameters indicated that the polarity peak almost disappeared due to the disturbed flow. Therefore, Rac could not be activated in a polarized way upon disturbed flow, and the polarity of Rac should be related to the exact flow direction on the cell surface under flow.

**Table 1:** The parameters from fitting curve in different groups.

	|A_1_-A_2_|	X’	Slope
Control	1.1476±0.4368	12.3000±5.7108	-0.2946±0.2286
Disturbed Flow	0.4075±0.1738*	24.2500±12.3879*	-0.0702±0.0230*
ML-7	1.2948±0.8423	17.0000±4.4136	-0.2241±0.1414
CytoD	1.3153±0.8565	31.5000±15.9123*	-0.1330±0.0860
NOCO	0.3086±0.1357*	30.0000±12.4685*	-0.0523±0.0462*
CHO	0.2640±0.1574*	22.7500±11.5660*	-0.0070±0.0734*
BA	2.3877±1.5229*	15.16831±5.27111	-0.1869±0.1007

(*p<0.05 compare to control)

### Cytoskeleton relates to Rac polarity

**Fig 4:**
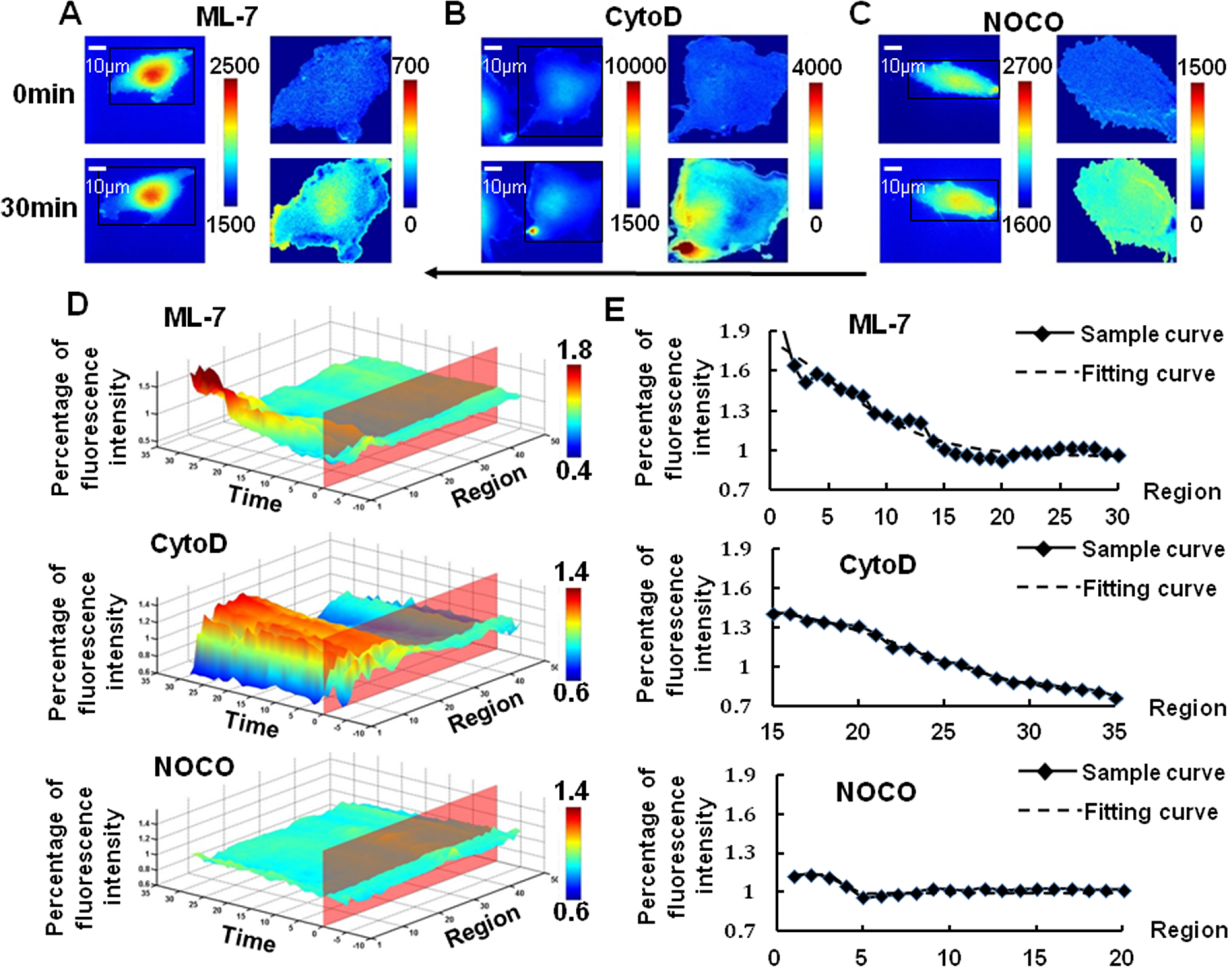
Shear stress caused a polarized Rac activation in BAECs after pre-treatment of ML-7 and CytoD but eliminated by NOCO. The images of BAECs transfected with biosensor (left panel) pretreated with ML-7(A, n = 7), CytoD(B, n = 6) or NOCO(C, n = 8) upon laminar flow application were dealt by the imaging analysis(right panel). The 3D spatiotemporal map (D) of averaged single cell images showed an obvious shear stress-induced Rac activation at the downstream of BAECs from ML-7 (Above) and CytoD group (Middle), but not NOCO (Below). After that, the data of the thirtieth minute from three groups were fitted by logistic curve. Above was the ML-7 group, middle was CytoD and below was the NOCO group (E). The arrow indicated the main flow direction.

Since cytoskeleton plays a critical role in providing mechanical support for the plasma membrane [18], its role in flow-induced Rac polarity has also been explored. BAECs were pretreated with 5 μmol/l of myosin light chain kinase (MLCK) inhibitor ML-7[12, 19], which could only eliminate the force transmission through microfilaments while the structure could remain intact, for 1 hr before laminar shear stress application. The results in Fig 4 showed that the Rac activation still appeared in a polarized manner, and the logistic parameters indicated that ML-7 only had gentle effect on the intensity or slope of Rac polarity(p>0.05).

After that, 2 μmol/l of Cytochalasin D (CytoD), incubation for 1 hr, was used to destroy the structure of microfilament in BAECs. After 30 min of laminar shear stress application, the course of Rac activation at downstream along the flow direction was not much affected by CytoD, while the parameter X’ increased obviously(p<0.05), indicating that the Rac polarity moved away from the downstream (Fig 4, Table 1).

However, a visible suppression of Rac polarity could be observed at the downstream of the cell upon being dealt with 1 μmol/l of nocodazole (NOCO), a depolymerizing agent of microtubules, for 1 hr of incubation before laminar shear stress application (Fig 4). The fitting result showed that the |A_1_-A_2_| slumped obviously(p<0.05), indicating that the amount of activated Rac at downstream was reduced by NOCO upon laminar flow. The parameter X^’^ migrating and slope reducing obviously(p<0.05), indicating that activated Rac distributed evenly across the whole cell. It was clear that Rac activation disappeared when microtubules were destroyed.

### Changing cell membrane fluidity alters shear stress induced-Rac polarity

**Fig 5:**
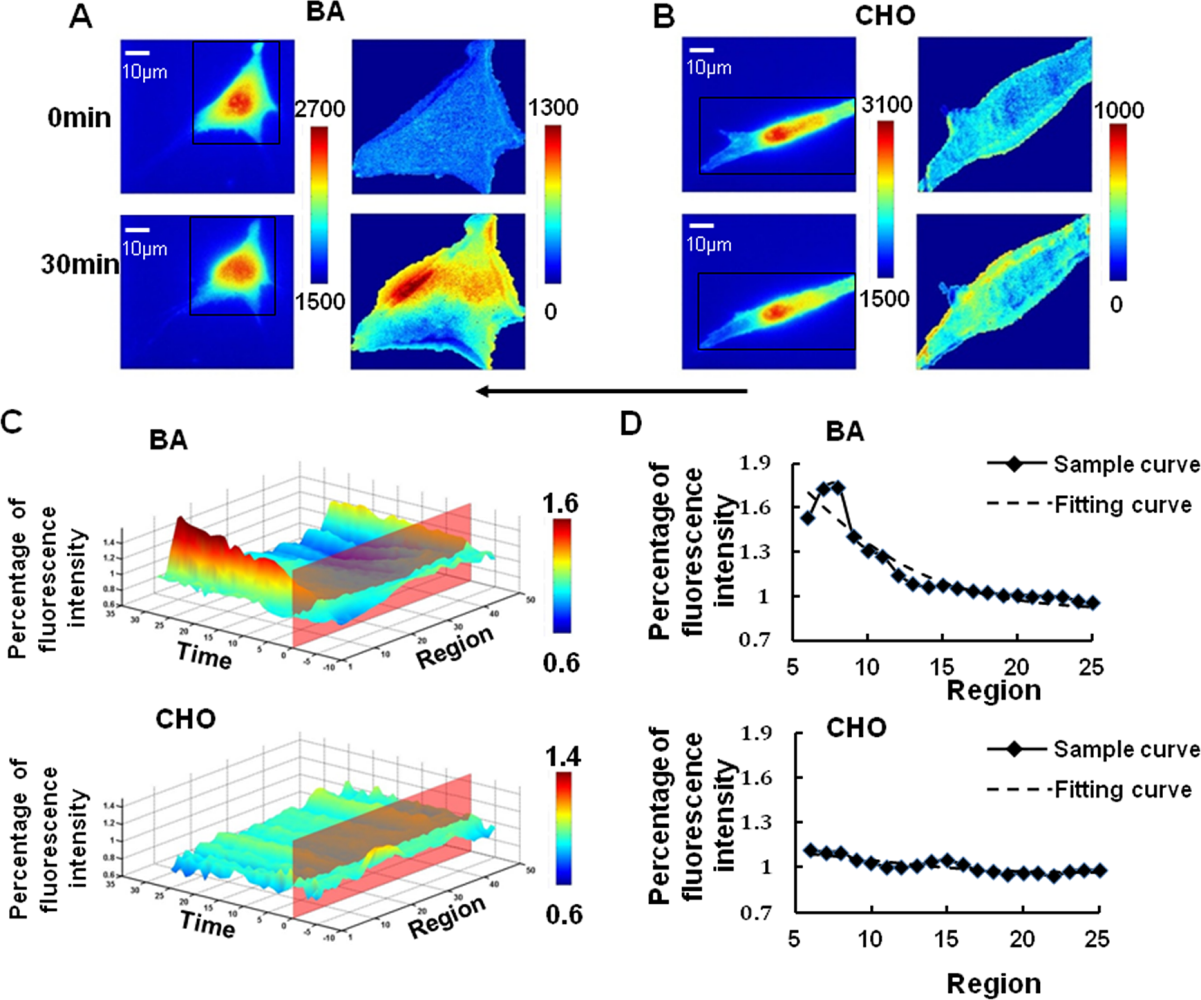
Shear stress caused a polarized Rac activation after pre-treatment of BA but eliminated by CHO. The images of transfected BAECs (left panel) pretreated with BA (A, n = 5) or CHO (B, n = 5) upon laminar flow application were dealt by the imaging analysis (right panel). The 3D spatiotemporal map (C) of averaged single cell images showed an obvious shear stress-induced Rac activation at the downstream of BAECs from BA (Above) group, but not CHO group (Below). After that, the data of the thirtieth minute from BA group (Above) and CHO group(Below) is extracted and fitted by logistic curve(D). The arrow indicated the main flow direction.

In our further experiment, 0.1 mmol/L of Cholesterol (CHO) for 3 hr or 45 mmol/L benzol alcohol (BA) for 45 min of pre-incubation were applied before laminar shear stress to reduce or enhance the fluidity of cell membrane, respectively[19, 20]. The result of CHO treatment showed no obvious deformation of the cells under shear stress compared with control group. However, the level and slope of shear stress-induced Rac activation both were affected by CHO. Upon laminar flow, after CHO pre-treatment, little Rac could be found activated at the opposite side facing the flow, and the Rac polarity activation was suppressed more when the stiffness of cell membrane was increased. It was obvious that CHO had an inhibitory effect on the overall level of Rac activation and the shear stress-induced Rac polarity (Fig 5). Conversely, the result of BA pre-treatment enhancing the fluidity of cell membrane showed an aggravated deformation of cells. The fitting analysis showed an earlier and more obvious polarity peak of Rac upon shear stress application (Fig 5, Table 1). These results demonstrated that cell membrane fluidity had an evident effect on the Rac polarity definitely.

## Discussion

### The Rac is activated in a polarized manner by shear stress and inhibited by disturbed flow

In this paper, the different flow pattern-induced local activation of Rac has been investigated in BAECs using a genetically-encoded PAK-PBD-GFP reporter, as well as the effects of various inhibitors in flow-induced Rac polarity. It has been proved that PBD can bind the activated GTP-bound forms of both Rac and Cdc42 in vitro, while its GFP-tagged counterpart (PAK-PBD-GFP) mainly detects activated Rac rather than activated Cdc42 in living cells[13–15]. Depending on this feature, the biosensor can only show the localization of activated Rac. In addition to the experiments, we have established a calculation method to analyze and visualize the subcellular distribution of molecular activity based on imaging analysis and Logistic curve fitting. Three-dimensional images containing the spatial and temporal course were used for the whole visual representation. After 30 min of shear stress application, the 3D images showed that the Rac polarity became steady. Hence the image of the thirtieth minute was chosen as fitting data which should be a good description of shear stress induced-Rac polarity.

Although the appearance or disappearance of Rac polarity could be observed directly in 3D figures, the mathematical model (1) made it more accurate and objective to measure the level of Rac polarity. The parameters of the Logistic curve represented different meanings as discussed earlier in the paper. |A_1_-A_2_| decreasing or even disappearing implied that the intensity of Rac activation declines. The subcellular position of activated Rac could be measured by X’ and D was a parameter related to the slope of the fitting curve. When the activated speed of Rac changed, the slope calculated from D would be changed. With this method of imaging analysis and the four parameters of Logistic curve fitting, we can easily evaluate the shear stress induced Rac polarity comprehensively.

Although Rac would concentrate in the downstream region of ECs upon shear stress treatment [9], which is a course containing a transient increase, a peak and a decline down to basal level [21], there is no clear evidence on, how does the disturbed flow affect subcellular Rac translocation? Consistently, our results also showed an obvious local activation of Rac at the downstream edge of BAECs along the flow direction after a relative steady phase. However, flow with irregular directions could not trigger the polar distribution of activated Rac. The quantitative results confirmed the experimental phenomena, since all the parameters obviously were differences between the group of laminar and disturbed flow, which would be the reason why disturbed flow could affect the cell migration and arrangement.

### The laminar flow-induced Rac polarity is based on cell membrane fluidity

It has been reported that nuclear bulge, caused by laminar flow, increases the tension in upstream region, thus the cell membrane fluidity will be increased in a polar manner [21]. Moreover, the apical cell membrane enduring a direct perturbation reflected in membrane fluidity would help cell to distinguish different temporal shear gradients [20]. Hence, we postulate that the directional shear stress-induced asymmetric change of cell membrane fluidity will impact on the Rac polarized activation. Indeed, laminar fluid shear leads to temporally varying and spatially heterogeneous stresses on the cell membrane [20], which has also been proved by our previous report [19], and then causes cell deformation along the flow direction, resulting in an increase in lamellipodia protrusion along the flow [22]. The formation of lamellipodia in the direction of flow is fast and structurally identifiable, which is an essential process in formatting focal adhesion to support friction force for cell migration [7]. Based on our further results of logistic curve fitting, when the membrane fluidity was inhibited, the decline degree of activated Rac was reflected by the distinct reduction of |A_1_-A_2_|, and the slope was much smaller than the control group, indicating that there was no outstanding polarity peak. On the contrary, the intensity of Rac polarity increased, after softening the membrane and enhancing the fluidity. This may be due to the reason that softer cell membrane becomes much easier to deform, and the directional shift of lipid rafts will be stronger under the shear stress, which may leads to stretch of cytoskeleton alone the direction of stress and then the higher activation of Rac at the downstream; the harder membrane is more resistant in deformation when shear stress is applied to BAECs, which leads to the decline of flow-induced polar activation of Rac. While under disturbed flow without apparent direction, the local difference of cell membrane fluidity and the shift of lipid rafts will be eliminated, which cannot result in the polar Rac activity.

### The laminar flow-induced Rac polarity is mediated by microtubules

Since cell membrane and the bound cytoskeleton on some sites constitute a complex network [23], stress waves on the membrane could be transmitted via cytoskeleton [24]. It’s reported that when ECs are exposed to sustained shear stress with a clear direction, the cytoskeletal fibers undergo remodeling by staining of cytoskeleton proteins such as actin, tubulin and vimentin, and become oriented along the direction of shear flow with a consequent alignment of the cell [25]. But under disturbed flow, the cytoskeletal fibers and the cell orient randomly similar to that in static condition. It seems that the whole cell polarity, including the Rac, should be the result of cytoskeletal fibers remodeling upon flow pattern stimulation. Indeed, shear stress induced-Rac activation depends on cytoskeletal integrity [26], which is also verified by our results. Furthermore, our finding showed that shear stress induced Rac activation promptly, which is consistent with the previous report [26]. This force transmission is too rapid for rearrangement of cytoskeleton and should happen before the rearrangement. Therefore, according to our results, cytoskeleton is associated with the planar polarity of shear stress force transmittion along cytoskeleton, but a great possibility also exists that cytoskeletal arrangements, caused by local force transmittion, and could contribute to Rac polarity in turn.

However, which component of the cytoskeletal fiber plays more significant roles in shear stress-induced Rac polarity is still elusive. Some experiments suggested more important role of filament system, while the microtubules maybe just changed passively to play gentle roles in the process of stress-induced cell polarity [27, 28],while microfilaments provide driving force in migrating cells by actin polymerization [29], and actin alignment is also an essential biological processes in Rac activation upon shear stress [30]. However, there was also a different mechanism considering that microtubule should be the major structure which transduced the stress to activate the local Rac of the far end of the cell upon local stress application [24]. Since microtubules were reported growing into the downstream edge contributes to Rac1 activation and formation of short branched F-actin in migrating cells [31, 32], and disruption of microtubules affected the polarity of actin cytoskeleton [33, 34]. It is easy to understand that our experiments support a more decisive role of microtubules than microfilaments in shear stress-induced Rac polarity [24]. We also find that filament system participates in the process, and without the integrity of filament system, the intracellular stress from the flow could not transmit to the far end of the cell through microtubules only. Since destroying microfilaments will also change its anchoring function to the lipid rafts in kinetic features[35], and Rac associates with membranes in lipid rafts primarily[36], filaments will also affects the manner of Rac polarization probably. However, the exact reason which causes this difference still needs to be deeply elucidated in the future.

### The model of shear stress-induced Rac polarity

Force applied to the cell membrane led to the deformation of upstream region directly, but Rac was activated at the downstream periphery. How was the effect of shear stress transmitted from the upstream region to the other side, and transduced into biochemical activities in a process remained vague. Rac activation was eliminated during microtubules depolymerization or the decrease of cell membrane fluidity, while the activated manner and duration of Rac were changed by microfilament depolymerization. These evidences suggested that shear stress induced-Rac activation was depending on the interaction between membrane and cytoskeleton. A local force applied on the cell membrane could be transmitted along the cytoskeletal network to induce Rac activation on the sites cytoskeleton forming strong connections to the plasma membrane [26]. This interaction probably involved the cytoskeleton deformation caused by local deformation of cell membrane and the cytoskeleton in turn regulating cell membrane fluidity. The two components, membrane and cytoskeleton, are required.

**Fig 6:**
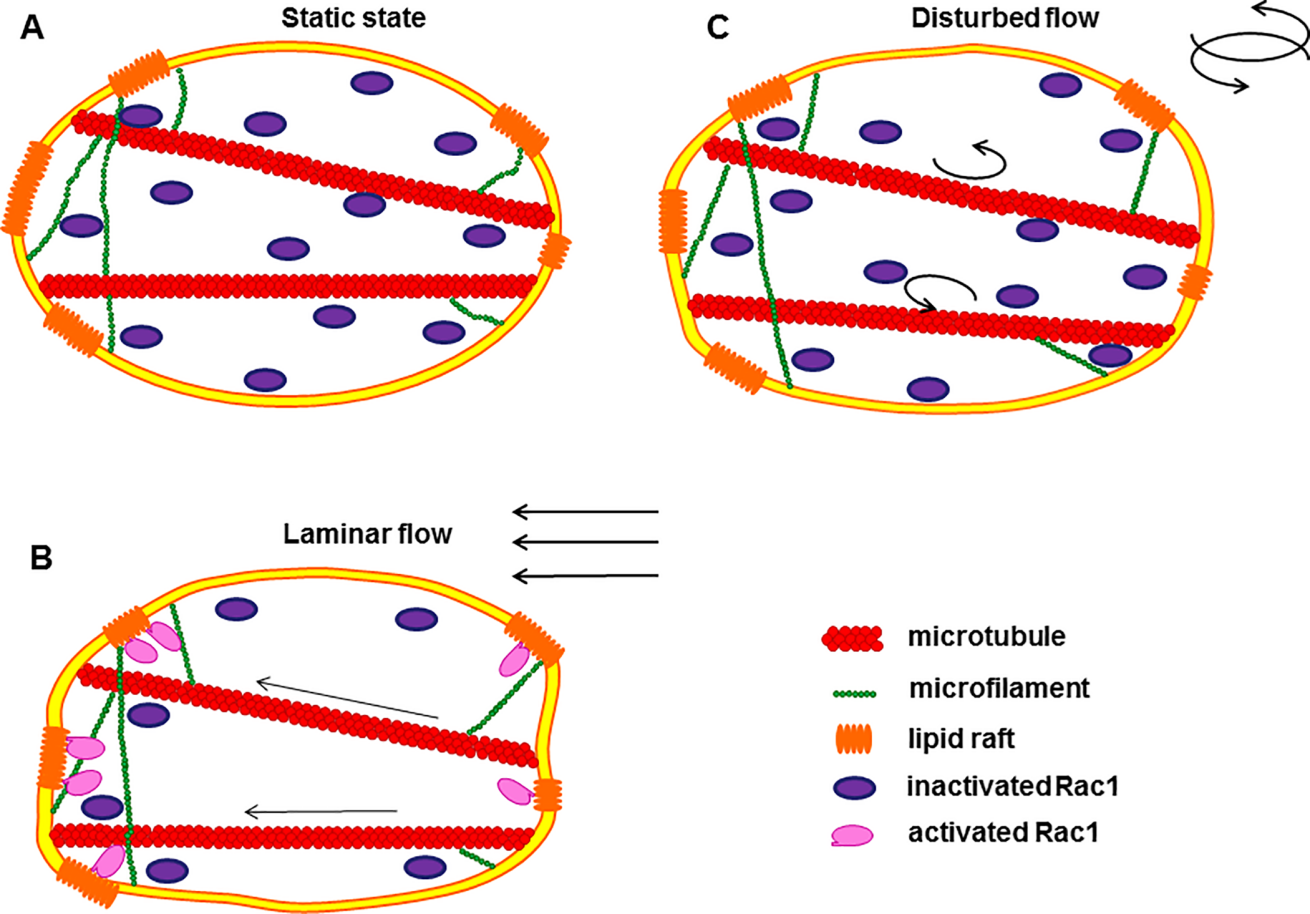
The model of Rac activation under laminar flow and disturbed flow. (A) The static condition before shear stress application. (B)Under laminar flow, cell membrane deformed along the flow and transmitted force to the downstream edges of cells with the help of cytoskeleton, especially microtubule, and then active Rac.(C)While under disturbed flow, the cell membrane and cytoskeletal fibers changed randomly without any obvious spatial direction.

In summary, although the disturbed flow inhibits the shear stress induced Rac activation, the mechanism still could be discovered by changing experimental conditions under laminar flow. Therefore, the model of shear stress-induced Rac polarity can be illuminated (Fig 6). When mechanical forces with clear direction loading, ECs perceive the spatial pattern of force and deform along the flow because of the change of plasma membrane fluidity. Force is being transmitted to the downstream edge of cells rapidly and cytoskeleton network participate in this process, which is mainly microtubule binding to the membrane. Then the alternation of local strain at the downstream edge of cell promotes the polar Rac activation. But under the disorder of the direction and the magnitude of disturbed flow, the cell membrane should be changed randomly without any obvious spatial direction. The cytoskeleton fibers are not polarized in a clear direction and the cell orient is random. Therefore, force would not be transmitted to downstream to induce polar activation of Rac.

## Supporting information

S1 Code The code of analyzing Rac1 polarity. (M)

S2 Code The function code to make details of cell outline clear. (M)

S1 ZIP File Initial data of cells dealt with BA. (ZIP)

S2 ZIP File Initial data of cells dealt with CHO. (ZIP)

S3 ZIP File Initial data of cells in control group, part 1. (ZIP)

S4 ZIP File Initial data of cells in control group, part 2. (ZIP)

S5 ZIP File Initial data of cells dealt with CytoD. (ZIP)

S6 ZIP File Initial data of cells upon disturbed flow. (ZIP)

S7 ZIP File Initial data of cells dealt with ML-7. (ZIP)

S8 ZIP File Initial data of cells dealt with NOCO. (ZIP)
